# What Determines the Perception of Segmentation in Contemporary Music?

**DOI:** 10.3389/fpsyg.2020.01001

**Published:** 2020-05-28

**Authors:** Michelle Phillips, Andrew J. Stewart, J. Matthew Wilcoxson, Luke A. Jones, Emily Howard, Pip Willcox, Marcus du Sautoy, David De Roure

**Affiliations:** ^1^The Royal Northern College of Music, Manchester, United Kingdom; ^2^Division of Neuroscience and Experimental Psychology, Faculty of Biology, Medicine and Health, Manchester Academic Health Science Centre, The University of Manchester, Manchester, United Kingdom; ^3^Oxford e-Research Centre, Mathematical, Physical and Life Sciences Division, University of Oxford, Oxford, United Kingdom; ^4^Mathematical Institute, University of Oxford, Oxford, United Kingdom; ^5^The Alan Turing Institute, London, United Kingdom

**Keywords:** music perception, large-scale form, segmentation, contemporary music, Ligeti, live music

## Abstract

**Background:**

This study concerns the perception of musical segmentation during listening to live contemporary classical music. Little is known about how listeners form judgments of musical segments, particularly when typical section markers, such as cadences and fermatas, are absent [e.g., Sears et al. (2014)] or when the music is non-tonal (e.g., in much contemporary classical music).

**Aims:**

The current study aimed to examine the listeners’ segmentation decisions in a piece of contemporary music, Ligeti’s “Fanfares”?

**Methods:**

Data were gathered using a smartphone application [Practice & Research in Science & Music (PRiSM) Perception App] designed for this study by the Royal Northern College of Music (RNCM) Centre for PRiSM and the Oxford e-Research Centre. A total of 259 audience participants were asked to “tap” when they felt that a section had ended. Subjective responses were captured, as well as contextual data about the participants.

**Results:**

The audience members demonstrated high levels of agreement regarding segmentation, mostly at places in the music involving breaks in the musical texture (one piano hand resting), changes in dynamic (volume), and changes in register/pitch. A sense of familiarity with contemporary repertoire did seem to influence the responses—the participants who self-reported being familiar with contemporary music used a wider range of cues to make their segmentation decisions. The self-report data analysis suggested that the listeners were not always aware of how they made decisions regarding segmentation. The factors which may influence their judgment of musical segmentation are, to some extent, similar to those identified by music analysis (Steinitz, 2011) but different in other ways. The effect of musical training was found to be quite small.

**Conclusion:**

Whether musically trained and/or familiar with contemporary classical music or not, the listeners demonstrate commonalities in segmentation, which they are not always aware of. This study has implications for contemporary composers, performers, and audiences and how they engage with new music in particular.

## Introduction

When we listen to a piece of music, we perceive the information as consisting of separate units or events (Drake and Bertrand, 2001). Such a process may be referred to as grouping (Lerdahl and Jackendoff, 1983), chunking (Miller, 1956), or segmentation (Cambouropoulos, 2006). How listeners infer such chunks of information or segment the auditory stream may be influenced by multiple factors. These may include individual differences, such as musical expertise, and/or features in the music itself, for example, when the music changes significantly in some way (for example, in modality or tempo). Investigating how musical information is parsed in real time, i.e., what determines how one event is deemed to have ended and another begun, may grant valuable insights into the ways in which expertise may shape the experience of music and how some aspects in the musical surface or structure may be more perceptually salient than others.

Most popular studies and models of the perception of musical form (e.g., Meyer, 1956; Lerdahl and Jackendoff, 1983) are based on the perception of tonal music. Contemporary (or “new”) music is rarely tonal and hence does not include related features such as diatonic harmony, harmonic tension and release, harmonic closure (e.g., at the cadence), or melody as conceived in the conventional 19^th^-century sense. Tonal music is often constructed as a series of organized events existing in a hierarchy. However, this may not be the case for non-tonal music (Dibben, 1994).

Such models of music listening, although perhaps addressing tonal music most often, may have relevance for the perception of contemporary music. Temperley’s (2007) theories of music and probability not only examine tonal hierarchies in their discussions of patterns but also discuss how music perception relies on familiarity with musical structures (relevant for contemporary music) and the probabilities regarding which events follow which others. Other notable music scholars have theorized about musical form (Rothstein, 1991; Caplin, 2001; Marsden, 2005). However, such works of theory and analysis, while often discussing notions of perception and building on the work of Schenker (1979) and Lerdahl and Jackendoff (1983), rarely include non-tonal music as their focus and do not examine perception empirically. Notable scholars have taken non-tonal music as their focus, for example, Hanninen’s (2003) concept of “recontextualization,” particularly with regards to her work on Feldman’s music, Straus’s (2016) theories of pitch-class sets, and Chew’s (2001) work with mathematical models of perception of tonal music, and much of this work includes reference to how a listener may parse (or segment) musical information. However, again this literature takes a theoretical stance and does not seek to examine the perception of non-tonal music empirically.

A common assumption is that “atonal music is generally perceived as opaque and difficult to understand because it rarely makes aural sense to the uninitiated listener” (Spies, 2005). Such a stance raises the question of whether tonal and non-tonal music are perceived differently from one another, and if so, how? Also, to what extent might non-tonal music be more difficult to understand, and does this depend at all on expertise? Research regarding the perception of musical form and factors which may influence this (such as a listener’s level of musical training) has only recently begun to interrogate ways in which contemporary classical music is processed and stored (e.g., Schulze et al., 2012), and there are many unanswered questions in this field despite the obvious relevance to modern-day listeners, composers, and performers.

Over the last 30 years, multiple studies have explored the perception of musical form in tonal music. Most have suggested that local cues (small scale and short duration) may take priority over global relationships (large scale and overall formal structure) in the perception of musical information (Deliège et al., 1996; Tillmann and Bigand, 2004; Rolison and Edworthy, 2012). Moreover, studies which have examined the impact of rearranging segments of music on the listener have found that the participants may not be able to differentiate between the original and the rearranged pieces of music (Tillmann and Bigand, 1996) and that the listeners may not prefer an original over an arrangement (Eitan and Granot, 2008). Two studies by Granot and Jacoby (2011, 2012) suggested that the participants may demonstrate sensitivity to some aspects of musical form (e.g., the ABA structure, the placement of the development section, and features at the beginning and end of the work) but not to others (e.g., overall harmonic structure). In summary, this research on the perception of large-scale form suggests that global relationships in the structure of music may not be available to perception, or if they are, such perception may not be as prominent as the recognition of smaller-scale events.

The question of how such local events are parsed, or segmented, when listening to large-scale form has only recently begun to be explored. Empirical work has suggested that, in tonal music, cadences (Tillmann et al., 1998; Tillmann and Bigand, 2004) and long notes and rests (Bruderer and McKinney, 2008) influence the judgment of segmentation during music listening. Studies of non-tonal music are fewer. Clarke and Krumhansl (1990) undertook five experiments which investigated various ways in which trained and untrained listeners perceive musical form. Experiments 1–3 used Stockhausen’s *Klavierstueck IX* and experiments 4–6 used Mozart’s *Fantasie in C minor*, K. 475, as their stimulus set. The authors state that, overall, the results of these experiments suggest that the attempts to model the perception of large-scale form and predict the perception of segments (in this case, using the model proposed by Lerdahl and Jackendoff, 1983) may be successful such that the listeners largely agree on the segment boundaries (for both the works by Mozart and by Stockhausen) and that the listeners are largely accurate in judging the location of segments. These results were relevant when listening to both tonal and non-tonal stimuli, which the authors attribute to “the two pieces share[ing] some high-level property on which listeners focus” (p. 249). This study laid important groundwork for questions of perception of musical structure (including segmentation) in tonal and non-tonal music. However, the study also invited a more thorough investigation of the perception of contemporary music (the structure of Stockhausen’s *Klavierstueck IX* may not be representative of non-tonal music more generally or shares attributes with later contemporary music), with a larger participant pool representative of a broader demographic listening in an ecologically valid environment. The question of how segments are perceived in a live performance of contemporary non-tonal music is central to the current study.

Clarke and Krumhansl’s (1990) study used musically trained participants. There is varying opinion regarding whether listeners with musical training perceive musical structure differently from those with no training. Deliège et al. (1996) found differences, and Eitan and Granot (2008) found some evidence that musical training may lead to the preference of a hybrid version over an original. Phillips and Cross (2011) found that musically trained listeners, when asked to judge duration in retrospect, judged the length of an extract of tonal music to be shorter than those with no musical training. Ockelford and Sergeant (2012) suggested that musicians may process non-tonal structure differently due to their training; they asked 14 musicians to listen to tone-rows and rate the extent to which notes “fit” with the other notes (probe tone paradigm) and found that musically trained listeners may impose tonal frameworks on the perception of non-tonal music (however, the study does not include a non-musician group for comparison). In contrast, Tillmann and Bigand (2004) found that the participants prioritized local over global features in perception, regardless of musical training. Bigand (2002) conducted a series of experiments which explored the perception of melody and harmony and, finding no difference in perception between trained and untrained listeners, concluded that mere exposure to music in everyday life results in everyone being an “experienced listener” (p. 304). A review of studies which examined various ways in which music is processed (perception of tension and relaxation, link between theme and variations, expectation generation, locating local features in global structure, and emotional response) by Bigand and Poulin-Charronnat (2006) also found that music perception depends on exposure to music rather than on formal training. Hence, studies do not convincingly support claims that musical training may alter the perception of musical structure.

The exposure effect (Zajonc, 2001) has been demonstrated to be important in music listening. For example, listeners exposed to a new musical scale for 25–30 min may show “extensive learning as characterized by recognition, generalization, and sensitivity to the event frequencies in their given grammar, as well as increased preference for repeated melodies in the new musical system” (Loui et al., 2010, p. 377). The proposition in this study, that “knowledge of musical structure is implicitly acquired from passive exposure to acoustical and statistical properties of musical sounds in the environment” (p. 386), is important when considering the perception of contemporary music; how this is perceived may depend on the extent to which a listener has been exposed to a similar musical system, or grammar, previously. However, it may not be the case that a grammar may be assumed for contemporary classical music, and there is as yet no convincing evidence of such. It is therefore also necessary to consider empirical studies of learned familiarity with contemporary music. For example, Western listeners may acquire knowledge of the structure of non-Western music through exposure (Stevens et al., 2013), and both musicians and non-musicians may acquire knowledge of sequences of melodies through exposure to a new musical grammar (Rohrmeier et al., 2011). On the other hand, studies have suggested that non-tonal music may be more difficult to store in working memory than tonal music for both musicians and non-musicians (Krumhansl, 1979). Schulze et al. (2012) asked musician and non-musician listeners to indicate whether tonal and non-tonal sequences were the same or different from the ones previously heard and found that both musicians and non-musicians performed better for tonal sequences than for non-tonal ones. These studies suggest that not only may working memory be better for tonal than non-tonal music but also that this may be the case regardless of musical training. It is not clear whether these two theories—that non-tonal music may be processed differently or that processing depends on exposure—are competing or two sides of the same coin (i.e., that processing depends on the *extent* of the exposure). Given that it is also not clear whether those who listen to contemporary classical music might acquire a set of expectations based on a learned grammar, this seems to be an important research question to pose in the current study.

Perception of structure in music relies on the way in which information is grouped and stored in memory or segmented. Moreover, the way in which musical information is organized and structured also plays a significant role in how that information is processed and remembered (e.g., Lerdahl, 1992 and notions of tonal pitch space, and probe tone and tonal hierarchy experiments such as Krumhansl and Kessler, 1982). Experimental work has recently begun to explore the sense of segmentation in contemporary music. Hartmann et al. (2017) asked 18 musicians and 18 non-musicians to listen to six 2-minute stimuli of unfamiliar music and to note segment boundaries (which they define for the participants as “instants of significant musical change,” p. 6) by pressing a computer keyboard space bar. In a second experiment, the same 18 musicians were invited to listen and mark instants of significant change once again and then to revisit the places they had marked and move these as they wished after the second occasion of hearing. They then rated the strength of each change that they had marked. The study indicated that musical training may play a role in the perception of segmentation.

The question of what constitutes a “significant change” in music perception is an important one. Models of segmentation have highlighted relevant factors including emotion (Aljanaki et al., 2015), rhythm, timbre, and harmony (Jensen, 2007), and novelty (Foote, 2000). However, such modeling exercises have largely sought to represent experiences of tonal music (for example, Foote, 2000 discusses a change of tonality as representing a significant change). Most of these musical factors are relevant to contemporary music listening, but few studies have sought to explore how these may lead to the perception of change in non-tonal music.

The participants in all studies discussed so far listened to stimuli in a laboratory or office environment. Only recently have studies begun to collect data in a live concert hall setting, which could be considered a more ecologically valid setting. As Broughton et al. (2008) note: “There is a need to understand how audience members respond to a concert as the music unfolds in time” (p. 366). A live performance may be considered more immersive than listening to a recording, involving auditory and visual information and a close focus on the performance as the main object on which attention is focused. Indeed the results of the study by Broughton et al. (2008) do suggest that audience response is partly based on the bodily movement of the performer (in this case, a solo marimba player). During a concert hall performance, the listeners have usually elected to listen attentively to music. Sound quality is also likely to differ between a live concert half performance and a recording, with the latter preserving more of the detail of the acoustic sound (without recording, editing, and altering this and without the sound being passed through headphones or speakers or listened to in an otherwise noisy environment). It could thus be deduced that a live music listening experience may result in more attention being paid to the piece as it unfolds in time and to the chunks (or segments) of information in the auditory stream. Experiments conducted in a live concert hall environment may also further the understanding of what it means to be a performer or a member of the audience in a contemporary music performance in the modern day. A performance could be considered a co-creation of, in this case, the composer’s work, a pianist, and the audience as a collective (rather than a set of isolated individuals), in which relevant experiences include other audients’ reactions and the pianist’s unique interpretation and realization. An experiment in a live concert hall setting is therefore communal, and the liveness of the assessment is an essential part of it. The findings of such a study may aid the knowledge of how performers and venues may adapt programs to audience demographics:

The application of experimental techniques to the study of music performance in its own environment builds new pathways for performing musicians, teachers, researchers, and those involved in the presentation of music performance to better understand the behavior and development of audience members. Such research in the future will be of great interest to the aforementioned groups and impact upon the creation, presentation, and programming of live concert music. (Broughton et al., 2008, p. 369)

Egermann et al. (2013) asked 50 concert audience members to provide subjective responses to a live flute performance. In line with theories relating to the exposure effect, they found that the listeners’ expectations regarding musical structure were a strong predictor of emotional response. This study provides support for notions such as the “experienced listener” discussed above, i.e., that familiarity with, or exposure to, music may influence the perception of segmentation and structure.

Results from relevant literature such as those studies discussed here suggest that there is a need for investigation of the experience of musical form as it unfolds in a live concert hall setting, where much of the real audience listening experience may be preserved (note that this method also involves limitations in the current study, which are discussed in the relevant section later). Furthermore, such investigation needs to advance the understanding of music created today rather than of tonal music composed in previous centuries. Studies should aim for data gathered from larger audiences, in excess of 50 participants, which is the maximum in existing studies. Finally, data on environmentally valid listening experiences should be gathered *via* means which are familiar to listeners and as unobtrusive as possible on the listening experience. This study seeks to address these gaps in knowledge in this field.

## The Current Study

The aims of this study were to examine how audience members perceive segmentation in a live performance of contemporary classical music and whether perception varies according to musical training and/or familiarity with contemporary music in general. The study sought to address the gaps in existing empirical work discussed above by employing a smartphone app designed for the study, which allowed live concert hall audience participants to tap their devices when they considered a segment to have ended.

### Research Questions

1. Is there evidence of agreement among audience members about segment boundaries in a piece of contemporary music?
2. What musical and sonic features, if any, occur where there is agreement on segment boundaries?
3. Does familiarity with contemporary music influence the decisions relating to segmentation?
4. What musical and sonic features do listeners self-report to have guided their decisions regarding segmentation and to what extent do these match the empirical tapping data collected?
5. Does musical training influence the decisions of segmentation?

## Materials and Methods

### Ethical Considerations

The study was granted ethical approval by the Royal Northern College of Music (RNCM) Ethics Committee (REC 131, approved 13 September 2017). The first screen of the app contained information relating to ethical consent and details of how the data would be processed, stored, and used.

### Participants

The 259 participants were audience members who attended an evening concert event in October 2017 as part of the Manchester Science Festival (MSF), which is run annually by the Science and Industry Museum in Manchester. The event was advertised in MSF publicity in print and online and in the event brochure of the RNCM, where the event took place. Those who purchased tickets for the event were subsequently contacted by email and invited to download the Practice and Research in Science and Music (PRiSM) Perception App (free to download *via* Apple and Android application online stores) in advance of their attendance at the event. Participation in the study was optional, and the audience members could attend the performance without taking part in the study. The 259 audience members who did opt to take part represented 43.2% of the 600 total attendees at the event. The performance was part of the launch event for the PRiSM research center titled “The Music of Proof: What Does Maths Sound Like?” Audience members included musicians from the RNCM and members (both adults and children) of the general public with an interest in music, maths, or science communication (as the event was part of a science festival).

Expert musicians were those that reported having had 10 or more years of musical training (52 participants in total). Those familiar with contemporary classical music were considered to be the participants who gave a response of 5, 6, or 7 to the question “As a listener, how familiar are you with twentieth-century music?” and those unfamiliar were the participants who responded with 1, 2, or 3. Although there was some overlap in these groups (those with musical training and those familiar with contemporary classical music), the overlap was only partial (46.67% of the participants who responded with 5–7 on a seven-point Likert scale to the question regarding their familiarity with contemporary classical music were also considered to be musicians for the purposes of this study, i.e., they had 10 or more years of musical training).

### Apparatus

The audience responses were captured on the participants’ own smartphones or tablets *via* the PRiSM Perception App, which was designed for this study. The source code used for the mobile app is registered under DOI “10.5281/zenodo.2542790”. Each device was used by one person and data were captured in real time.

After an “about this app” screen (described above and which included the ethics statement), the app consisted of three pages on which the participants were asked to enter data:

1. “Your profile” (date of birth, musical and mathematical training and experience, education, and how often the participants listen to music).
2. “Performances” (including the button to tap in response to the live performance).
3. “Questions” (questions relating to the experience of the stimulus).

### Stimulus

The participants heard the solo piano piece “Fanfares” from *Etudes* (Book 1, 1985) by Ligeti. The piece remains at a consistent tempo throughout, and while one piano hand plays quavers (seven quavers per bar), the other plays a motif often referred to as “horn fifths” or a variation on this motif. The material switches between the piano hands and changes in register and dynamic (volume) throughout the piece (although these changes are less overt in the second half of the piece). Although occasionally one piano rests for a number of quaver beats, there is never a break in both piano hands until the final bars of the piece. Thus, the work could be seen as being made up of 8 to 10 bar “phrases,” but the motion is never interrupted completely, and those segment boundaries commonly found in tonal music (cadences, fermatas) are not present. Steinitz’s (1996, 2011) expert analysis of the work focuses on the first 45 bars and outlines how the motif changes hands every 8 to 10 bars, with the accompanying ostinato being played 208 times in the duration of the piece.

This contemporary study for solo piano was performed in a large concert hall in front of 600 audience members, of which 259 opted to take part in the current study. The work was selected for the experiment for the following reasons:

1. As a composer, Ligeti is firmly established as representative of contemporary composition.
2. Ligeti’s *Etudes* is considered an important part of contemporary music repertoire and is regularly performed.
3. “Fanfares” includes features common to contemporary music (multiple series of notes which repeat, including an ostinato pattern, and a recurring motif commonly referred to as “horn fifths”).
4. “Fanfares” lacks many of those features commonly found at points considered to mark section boundaries in tonal music (silences, harmonic closure, and changes of meter or tempo).

The live performance of this work lasted for 3 minutes and 26 seconds (i.e., 206 s).

### Procedure

The participants could opt to fill in the “About You” page of the smartphone app before they arrived at the performance or afterward (it was compulsory to complete this page on the app in order to proceed to the experiment page). Immediately before the performance began, the participants were advised to take out their devices and open the “Performances” page on the PRiSM Perception App, which asked them to wait for the instruction to begin the participation in the study. A countdown was then given to the participants by a member of the PRiSM research team, at the end of which they were instructed to tap their screen to synchronize the devices to the timings of the performance. The performance then began, and the participants tapped the green button displayed on the app whenever they felt that a section had ended. The participants could also tap a button to mark the previous tap as an “error.”

Following the experiment, the participants were asked to respond to a final series of questions concerning their experience of the piece (on the “Questions” page of the app), including their enjoyment of and familiarity with the piece, familiarity with contemporary music as a genre in general, and how they made decisions regarding section boundaries. A full list of the questions can be found in Appendix 1.

### Analysis

The following steps were used to analyze the data:

1. A chi-square goodness-of-fit analysis was performed to examine whether the total taps were equally spread across the performance or whether they were not (research question 1).
2. The taps were divided into 2-s windows, i.e., the taps were grouped into clusters of 2 s each throughout the duration of the piece. 2 s was chosen as the relevant size of the groups as each bar in the performance lasts approximately 2 s, and the maximum size of one of the “breaks” (where one piano hand rests for a number of quaver beats) is 2 s. Therefore, 2 s is the upper limit for where we would expect audiences to identify a new segment. Additionally, comparable empirical studies have used windows [termed by Broughton et al. (2008) as a “lag,” which they defined as 1.5 s for the purpose of their study] varying between 0.5 s (Geringer, 1995) and 3.25 s (Krumhansl, 1996). 2 s is therefore within the normal range established in the field when examining how long after an event a participant can be considered to have responded to that event.
3. The top five 2-s windows where the participants tapped were examined to explore which musical features occurred at these points (research question 2).
4. The top 10 2-s windows where the participants tapped were examined for those classified as familiar and not familiar with contemporary music (research question 3).
5. The participants’ self-reports of how they decided where to tap (part of the post-performance “Questions” page of the app) were analyzed to determine what the most common factors were in guiding the participants’ decisions regarding segmentation (research question 4).
6. The results of step 5 above were used, along with relevant research, to construct a new prediction of where the participants may tap.
7. This new prediction was compared to the actual groups of taps, including an investigation of the relevance of this new prediction to musicians *vs*. non-musicians (research question 5).

## Results

**FIGURE 1:**
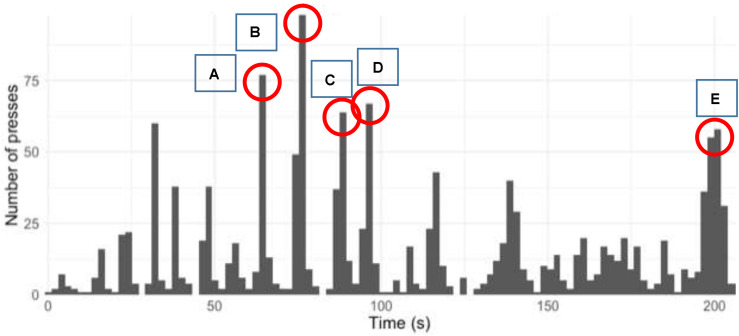
All taps (“presses”) by all participants during the performance of the work, split into 2-s windows (capital letters A–E refer to the aspects of this analysis which will be referred to in the outline of the results of the study).

The 259 participants tapped an average of 8.16 times (standard deviation: 4.43) during the 3.78-min performance (minimum taps: one, maximum taps: 21). The taps which the participants marked as “errors” were removed prior to analysis. Figure 1 shows all the taps by all the participants during the performance of the work, split into 2-s windows.

A chi-square test of goodness-of-fit was performed to determine whether the taps were spread evenly over the duration of the performance. The analysis suggested that the taps were not equally distributed across the total duration of the performance, χ^2^(36) = 543.81, *p* < 0.0005.

The five most common 2-s windows where the participants tapped occurred at the following points in the musical work (letters A–E below refer to the labels in Figure 1):

– A—bar 63, break of eight quavers in one hand, followed by a change in register and dynamics (*ppp* to *ff*) in the right hand.
– B—bar 74, break of 12 quavers in one hand, followed by a significant change in register in the left hand.
– C—bar 88, break of 10 quavers in one hand, followed by a change in register in the left hand, and dynamics in the left (*pp* to *ff*) and the right hand (*pppp* to *mp*).
– D—bar 96, break of 10 quavers in one hand, followed by a change in register in the left hand, and dynamics in the left (*mp* to *pppp*) and the right hand (*mp* to *pp*).
– E—bar 210, penultimate note (semibreve) of the piece.

From these five points, the following observations may be made: the factors which governed the decisions in relation to segmentation for all participants include: a break (quaver rests) in one piano hand, a change in register (or pitch), and a change in dynamic (or volume/listening level). The listeners also marked a segment break at the end of the piece when the consistent motion of the notes is replaced by one long note.

If points A–E above are reordered with the point which received the highest number of taps first, the following list results:

1. B—bar 74, break of 12 quavers in one hand, followed by a significant change in register in the left hand.
2. D—bar 96, break of 10 quavers in one hand, followed by a change in register in the left hand, and dynamics in the left (*mp* to *pppp*) and the right hand (*mp* to *pp*).
3. A—bar 63, break of eight quavers in one hand, followed by a change in register and dynamics (*ppp* to *ff*) in the right hand.
4. E—bar 210, penultimate note (semibreve) of the piece.
5. C—bar 88, break of 10 quavers in one hand, followed by a change in register in the left hand, and dynamics in the left (*pp* to *ff*) and the right hand (*pppp* to *mp*).

This reordering could suggest that a break (quaver rests) in one piano hand is the most common factor in leading the listeners to perceive segmentation.

**FIGURE 2:**
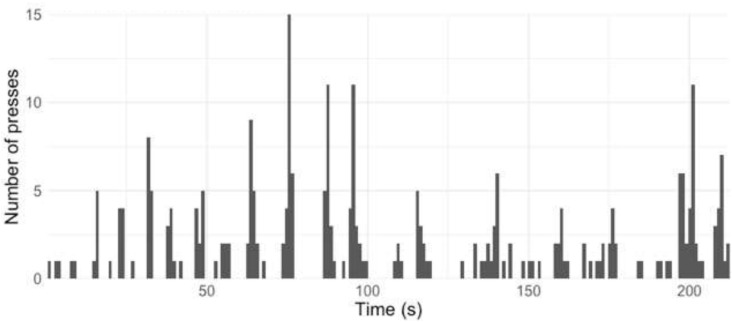
All taps (“presses”) by all participants familiar with contemporary music, split into 2-s windows.

**FIGURE 3:**
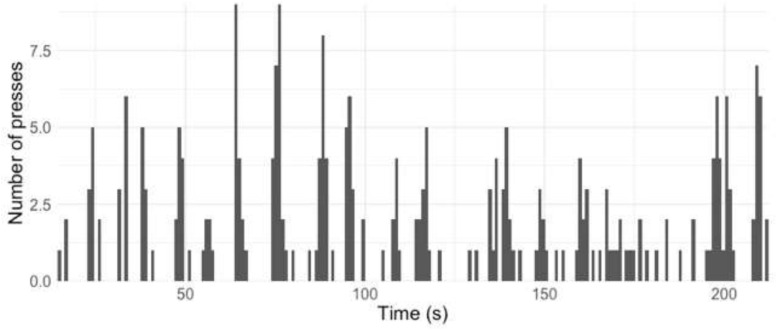
All taps (“presses”) by all participants not familiar with contemporary music, split into 2-s windows.

Figures 2, 3 give the equivalent visualization in Figure 1 of taps in 2-s windows for those who self-reported as being familiar (Figure 2) or not familiar (Figure 3) with contemporary music.

As the participants filled in this page of questions (which included familiarity ratings) after the performance, this gave the participants the option of not completing these questions. A total of 85 out of the 259 participants who provided tapping responses completed these questions. The method of analysis of these familiar and not familiar groups was informed by this fact, i.e., not all participants whose data features in Figure 3 responded to this part of the smartphone app.

The top 10 points where all the participants, familiar and not familiar with contemporary music, tapped are outlined in Table 1.

**TABLE 1:** Top 10 points (in seconds) where all participants, familiar and not familiar with contemporary music, split into 2-s windows, tapped (the “Notes” column refers to the comments in the results section).

Condition (familiar *vs*. not familiar)	Time point in the duration of the piece (s)	Number of taps in this 2-s window	Notes
Familiar	32	8	6
Not familiar	64	9	3
Familiar	64	9	3
Not familiar	75	7	7
Familiar	76	15	1
Not familiar	76	9	1
Familiar	88	11	2
Not familiar	88	8	2
Familiar	96	11	4
Familiar	201	11	5

From this table, it can be seen that the familiar and the not-familiar groups both tapped at the main points at 76 s (“1” in the notes column in Table 1), 88 s (“2”), and 64 s (“3”). There is hence some agreement between these two groups on points of segmentation.

However, the remaining four points in Table 1 differ between the groups. First, the familiar group features six times in Table 1, compared with four times in the not-familiar group. This may suggest that those not familiar with contemporary music were in more agreement regarding segmentation with others than those in the familiar group. The remaining four points occur at the following points in the music (the numbers below refer to the “notes” column in Table 1):

4. In this bar, the music remains in the *pp* (right hand) and *pppp* (left hand) dynamic range (as for the eight bars before this), but the right hand changes register by moving upwards by c. three octaves.
5. This is the final bar of the music (two semibreves tied together).
6. In this bar, the music changes in dynamic (*mp* to *pp* in the left hand and *pp* to *f* in the right hand) and the two piano hands swap roles in terms of the musical information that they play—the right hand now plays the “melody” (“horn fifths”) material, while the left hand plays the quaver ostinato.
7. This is 1 s before the window at 76 s and is therefore most likely to be a marker of earlier responses to the same event.

Points 4 and 6 above suggest that there may be musical and sonic features which lead the listeners to mark a point of segmentation (change of register and change of musical material played by each piano hand) but that these may be detected more by the listeners who are familiar with contemporary classical music than those who are not.

In the post-performance “Questions” page of the app, the participants were asked to state in a blank text box the basis on which they made their decisions of segmentation. The responses were categorized into overarching themes, and the results.

The participants reported that the main factors used to make their decisions about where a segment ended were speed, register/pitch (a change in pitch or register), and a change in melody, theme, phrase, or motif. The work did not change tempo throughout but did include changes in various places in relation to pitch and melody. Any perception of a change of speed could not have resulted from changes in tempo in the music but may have been caused by changes in other musical characteristics.

Based on the self-reported data, it is possible to form new predictions regarding where the participants in the study may have tapped. Such predictions can also be informed by existing formal music analysis of the piece. At this point, it is important to acknowledge that all of these features, which may result in the listeners choosing to mark a segment boundary, may be thought of as distinct phenomena. On the one hand, the features may be the musical material itself, such as the melodic material, the notes themselves, and any moments where one hand of the piano has a break (rests). The second category of features could be termed the “sonic” features of the piece. Such sonic features include those aspects which may be used to determine auditory streams (in line with Bregman’s, 1990 theories of “auditory scene analysis”). These include changes in the quality of the sound, such as its pitch height (register) and listening level (or volume/dynamic).

The following segmentation analysis is based on the main musical and sonic characteristics which listeners self-reported to have guided their judgments of segmentation (see Table 2, which shows the main musical characteristics—other than “speed,” as the piece did not change tempo during the performance—as “melody,” “register/pitch,” and “dynamic”), plus segment boundaries identified by music analysis (note that the first five segments in Table 2 are also identified as the first five segments in the analysis of the work by Steinitz, 2011).^1^

**TABLE 2:** Prediction of segmentation in the piece based on participant self-reports of what musical features lead to segmentation decisions.

Segment number	Bars	Reason for production of segmentation identification (musical feature)	Number of taps at the beginning of this segment/end of the previous segment (see Figure 4)
1	1–8	Register/pitch	N/A
2	8–9	Break	6
3	10–17	Register/pitch	16
4	18	Break	21
5	18–26	Register/pitch + dynamic	17
6	26–27	Break	8
7	28–36	Register/pitch + dynamic	5
8	37–45	Register/pitch + dynamic	6
9*	46–53	Melody + dynamics	38
10	54–62	Register/pitch	18
11	63–73	Register/pitch + dynamic	13
12*	74–87	Register/pitch + dynamic	30
13*	88–95	Dynamics	30
14	96–113	Register/pitch + dynamic	26
15*	114–115	Break	37
16*	116–122	Register/pitch	29
17	123–129	Register/pitch	6
18	130–141	Register/pitch	3
19	142–170	Dynamic	9
20	170–177	Dynamic	18
21	177–end	Dynamic	5

As can be seen from the discussion above, the top five segments in which the participants tapped most commonly (at the beginning of the 2-s window) are 9, 15, 12, 13, and 16. The musical and the sonic features of these include a change in dynamics (or volume/listening level), a change in the kind of musical material which makes up the “melody” (i.e., a different motif to the “horn fifths”), a break in the musical material in one piano hand (rests), and a change in register/pitch. Given the balance of these features in these top five segments, the importance of each of these features in the perception of segmentation seems to be in the following order of priority:

1. Melody + dynamics.
2. Break in the musical material (one piano hand has rests).
3. Register/pitch.

A change in dynamics occurs at three of these top five segments and is the most common feature out of this list of three.

Segments 9, 15, 12, 13, and 16 (marked with an asterisk in Table 2) are the five segments identified in Figure 1 as being in the top five 2-s windows in which the participants tapped. The participants self-report that the main factors influencing their segmentation decisions are speed, register/pitch, melody, and dynamic. However, Table 2 suggests that these self-reports of which factors influence the decisions of segmentation are only partially accurate. The participants mainly appear to use dynamic, melody, a break in the musical texture (rests in one piano hand), and register/pitch to guide their decisions rather than changes in tempo.

**FIGURE 4:**
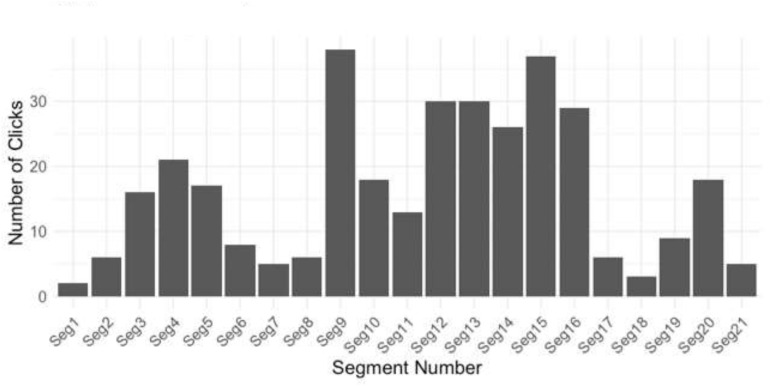
Number of taps (“clicks”) at each segment boundary outlined in the final column of Table 2.

These top five segments in Table 2 (9, 15, 12, 13, and 16) occur at bars 46, 114, 74, 88, and 116, respectively. This means that, when predictions of perceptual segmentation are formed using music analysis and self-report data regarding how the listeners make their decisions, these are the top five places where the predictions match the main points at which people tapped. However, the top five points at which all the participants tapped as per Figure 1, which shows the top taps when data are split into 2-s bins (rather than according to prediction of segmentation), occur at bars 63, 74, 88, 96, and 210. In summary, Figure 1 and Table 2 both have bars 74 and 88 in common, but they differ on their other top five points. This suggests that music analysis and self-report data alone do not account for how segmentation is perceived.

Logistic regression models were built to determine whether tapping (or not tapping) at a segment boundary was predicted by the degree of musical training that the participants had. We found that music training did predict whether people tapped at the segment 13 boundary (beta = 0.13308, *z* = 2.103, *p* = 0.0355) and at the segment 15 boundary (beta = 0.12626, *z* = 2.373, *p* = 0.0177), but not at any other boundary (all other *p*s > 0.05). As the level of musical training increased, the participants were more likely to tap at these segments. As can be seen in Table 2, segment 13 involves a change in dynamics, and segment 15 involves a break (one piano hand rests). This may be interpreted as suggesting that musicians and non-musicians rely differently on these cues for their judgment of segmentation, with musical training resulting in listeners being more likely to tap in response to some musical and sonic features. However, the effect of musical training is quite small as it only affected two of the segments, and there is no clear evidence that there are distinct features, or cues, at these points in the music. This should not, therefore, be considered a significant result.

All data and the script for the models discussed above are freely available at https://github.com/ajstewartlang/RNCM_Ligeti.

## Discussion

The research questions will be addressed in turn:

### Is There Evidence of Agreement Among Audience Members About Segment Boundaries in a Piece of Contemporary Music?

The results outlined above suggest that the taps were not evenly distributed across the piece of music (as can be seen clearly in Figure 1). We would have expected to see a more even distribution had taps been random. This suggests that there are commonalities in how segmentation is perceived by the listeners to this piece of music.

### What Musical and Sonic Features, If Any, Occur Where There is Agreement on Segment Boundaries?

The musical characteristics which influence such decisions include a break (quaver rests) in one piano hand, register (pitch), dynamic (volume), and iteration or breakdown of an established pattern (i.e., the end of the piece, when the continuous ostinato and melody pattern ceases). Of these, a break in the continuous quaver pattern in one piano hand seems to be the most common feature which leads to perceptual segmentation.

### Does Familiarity With Contemporary Music Influence Decisions Relating to Segmentation?

The results suggest that familiarity with contemporary classical music in general (not only with this specific piece of music) may influence the segmentation decisions when listening to this piece. More of the top 10 groups of taps, for those who self-reported being familiar with contemporary music or not, were found in listeners who were familiar. This suggests that these listeners pick up on a wider range of cues to make their decisions of segmentation. The features which resulted in the listeners marking a point of segmentation, such as change of register and change of musical material played by each piano hand, seem to be detected more by listeners familiar with contemporary classical music than those who were not.

### What Musical and Sonic Features Do Listeners Self-Report to Have Guided Their Decisions Regarding Segmentation, and to What Extent Do These Match the Empirical Tapping Data Collected?

The participants self-reported most commonly that speed influenced their decisions regarding segmentation. As this piece of music remained at one constant tempo throughout, this finding suggests that the listeners are not fully aware of how decisions regarding segmentation are made. The data analysis also suggests that the top five points at which the listeners perceived segmentation are different, depending on whether the data are analyzed in 2-s windows (Figure 1) or compared with a model of perceptual segmentation which was formed using self-report data and music analysis (Table 2).

Taken together, these findings indicate that the factors guiding the perception of a piece of music as it is realized in time may not be predicted by the listeners’ own accounts of perception or by an examination of the musical score. These data provide further support to the notion that the structures which may be relevant during an analysis of a musical score may not be the same as those relevant in the perception of the music as it unfolds in time (relevant debates in this area have concerned, for example, music and the golden section; see e.g., Cook, 1987; Phillips, 2019). Also, regardless of the level of musical training or familiarity with contemporary classical music, the listeners may not be able to entirely predict how a piece of music may be perceived. This has relevance for listeners, composers, and performers today.

One possible reason that the participants self-reported speed as playing a role in their decisions may be that speed was a proxy for a different musical characteristic. For example, perhaps *note density* (number of onsets in a given temporal window) was interpreted as speed. This interpretation proposes that a point where there are fewer notes in the bar, for example, where one hand of the piano does not play, could sound like a change in tempo. The results discussed in relation to research question 1 suggest that the listeners commonly marked a segment when one piano hand was resting and hence where there were fewer notes in the piece at the point. Perhaps note density is key in understanding the sense of segmentation during live music listening.

### Does Musical Training Influence Decisions of Segmentation?

Although the multiple logistic regression models used to analyze these data suggested that musicians were slightly more likely to tap at certain segments than non-musicians, this effect was found to be quite small. We therefore consider that there is no clear evidence for an effect of musical training on perceptual segmentation.

### Limitations

Data collection took place in a live concert hall environment, using a smartphone application which could be freely downloaded onto the participants’ personal devices. This allowed for high-resolution and non-obtrusive data capture, which may be conducted in a silent, low-light environment. Data collection by smartphone application also allowed for the participants to contribute responses both within (real-time responses to musical structure) and outside (demographic data and subjective responses to music) the concert hall environment. However, this element of the study design also resulted in some limitations to the study. Firstly, peripheral or direct vision of the other participants’ activity on the app could have altered behavior. It may have been the case that the listeners tapped due to seeing a neighbor tap, possibly demonstrating a desire to conform (Asch, 1951, replicated by Brandstetter et al., 2014). The results of the study by Brandstetter et al. (2014) suggest that the participants are even more likely to conform if a situation contains a high degree of uncertainty. The current experiment could be considered to include uncertainty, first, as it is not common that listeners are asked to respond to a concert hall performance in real time and, second, as conventional segment boundaries in classical music (silence, cadences, and pauses) were not available in this piece of music, and the decisions regarding segmentation may therefore have been difficult. However, not all audience members who attended the event took part in the experiment; 259 of the 600 audience members chose to download the app and take part in the study, i.e., 43%. The participants were not asked for their seat numbers and no data were collected on their physical clustering or dispersal. Those taking part in the study were not allocated particular seats apart from the non-participants. Therefore, it was not the case that all the participants were sitting next to someone else who was also participating. It is therefore unlikely that conformity could account for the main findings of this study.

A further limitation of this study was that, although the first research questions asked “is there evidence of agreement among audience members about segment boundaries in a piece of contemporary music?,” only one piece of contemporary music was used for the experiment. The results cannot be considered as generalizable to all contemporary classical music. However, the results may be relevant for considerations of perception of segmentation in music which does not contain any of those segment boundaries that have been shown to influence the perception in tonal music, and the method, app, and data analysis could be reproduced for comparative studies of future live performances.

The performance lasted for 206 s. This brings with it another limitation, which is that the participants’ responses may have changed over the duration of the listening period (e.g., they may have paid more attention to the performance at the beginning and therefore tapped more or less than in the middle). However, the taps at each of the segments outlined in Table 2 suggest that it is not the case that the listeners tapped less frequently in the second half of the piece than in the first half; there are 100 taps in the first 10 segments in this table and 115 taps in the segments in the second half. However, the cues to which the listeners responded may indeed have changed over the course of the work. Although Table 2 does not suggest this, changing responses over 206 s could have played a role in the results. Future studies could create multiple versions of the stimulus in which the segments are rearranged (similar to that of Eitan and Granot, 2008), and the listeners are asked to respond to segmentation in these new versions.

There are multiple different ways in which these data could have been analyzed. The analysis above addresses the research questions and leads to valuable insights which will hopefully pave the way for future studies. One aspect which could be examined in such studies includes the size of the temporal windows into which the tapping data were split. The current study used windows of 2 s due to the musical information in the piece, the rate at which events occurred, and existing standards in other relevant empirical work. However, future studies could consider different temporal windows.

## Conclusion

Overall, this study suggests that listeners do agree on segment boundaries when listening to this piece of contemporary classical music in a live concert hall setting as evidenced in their responses to segmentation in real time using a bespoke smartphone app. The segments which were identified by the participants seem to have occurred where one hand in the piano part had a break of 10–13 quavers (N.B.: some audience members could see the pianist’s hands and the piano keyboard, but only from a distance), where the music changed significantly in register/pitch, dynamic (volume), or where there was a change in the melody or motif (e.g., at the end of the piece). The musical factors which influenced segmentation were partly evidenced in the participants’ self-reports of how they made decisions, although speed was the most commonly reported reason for identifying change, and the piece was at a consistent tempo throughout. The perception of a change of speed may actually be a response to a change in note density. A break in one piano hand also leads to the perception of segmentation, which is a factor that could be linked to previous empirical studies of tonal music, which have also found that a rest, or break, provides a sense of a segment having ended. The marking of a segment boundary at a point where one piano hand rests could also be linked to the idea that the listeners perceive segmentation when there is a change in an aspect of the texture or timbre of the piece in general.

The results suggest that familiarity with contemporary classical music as a genre may influence the perception of segmentation. Those familiar with this genre seemed to use a wider range of cues to inform their responses (taps), including a change of register and a change of musical material played by each piano hand. These findings could be interpreted as supportive of the notion of the “experienced listener”—general music listening experience may play as significant a role in music perception as formal music training, or perhaps an even greater role.

The results of this study shed valuable light on how the listeners with varying levels of experiences of listening to contemporary classical music may parse musical information in real time in an ecologically valid setting. Such findings may be of interest to composers and performers who could make decisions regarding how they use musical material to suggest a particular structure to a listener (e.g., a performer could alter the dynamic level in different segments). However, given the results of the self-report data above, it is unlikely to be the case that composers or performers could predict all aspects of how segmentation may be perceived.

A further implication of these findings relates to concert hall programming and audience engagement. The perception of contemporary classical music may change as a listener becomes more familiar with this repertoire. Access to contemporary music may appeal to new audiences if these audiences can be gradually exposed to the musical and the sonic features of contemporary classical music, and it may not be the case that marketing should be targeted preferably at those potential audience members with formal musical training.

## Data Availability Statement

The datasets generated for this study are available on request to the corresponding author.

## Ethics Statement

The studies involving human participants were reviewed and approved by the RNCM Ethics Committee, Royal Northern College of Music. The patients/participants provided their written informed consent to participate in this study.

## Author Contributions

MP contributed to experimental design, literature review, data collection, data analysis, writing, review, and submission of the manuscript. JW contributed to experimental design and building of mobile phone software application. EH contributed to experimental design, data collection, and review of the final manuscript. MS contributed to experimental design, data collection, and review of the final manuscript. PW contributed to experimental design and review of the final manuscript. LJ contributed to data analysis and review of the final manuscript. AS contributed to data analysis and review of the final manuscript. DD contributed to experimental design, building of mobile phone software, application data collection, data analysis, advice on, and review of the final manuscript.

## Conflict of Interest

The authors declare that the research was conducted in the absence of any commercial or financial relationships that could be construed as a potential conflict of interest.

## Footnotes

1 It should be noted that, in the live performance which formed the basis for this study, the pianist skipped two bars (c. 2 s) of the original musical notation. The mistake was not audible, as verified during discussions with expert audience members (including Professor Richard Steinitz, author of Steinitz, 2011) but was detected during the analysis. This two-bar skip did not happen across any of the segment boundaries in Table 2 or at any of the points at which the audience marked on the main segment boundaries in the piece.

## Appendix 1—Survey Questions

Q1. What is your date of birth (YYYY-MM-DD)?

Q2. How many years of formal music training have you had (including A-level and any instrumental, vocal, or composition lessons)? [options: 0 1 2 3 4 5 6 7 8 9 10+]

Q3. Do you currently play a musical instrument, sing, or compose, and if so for how long? (Please select zero if you do not have any) [options: 0 1 2 3 4 5 6 7 8 9 10+]

Q4. How many years of formal mathematics training have you had (including A-level and any further study of mathematics)? [options: 0 1 2 3 4 5 6 7 8 9 10+]

Q5. Do you currently work in a field which requires mathematical skills, and if so for how long have you worked in this area? (Please select zero if you do not work with mathematics) [options: 0 1 2 3 4 5 6 7 8 9 10+]

Q6. What is your highest level of formal

Qualification? [options: GCSEs, A-levels, Bachelor’s Degree, Master’s Degree, Ph.D., other (please state in the text box)]

Q7. How often do you listen to music (of any style)? [options: never, occasionally, sometimes, most days, every day]

Q8. How long did you think that the piece of music lasted? [minutes and a seconds box, with the options that either can be left blank but not both]

Q9. Please describe the piece in three words [followed by a text box]

Q10. How did you decide when a section had ended [followed by a text box]?

Q11. How much did you enjoy the piece of music? (from 1 = I did not enjoy it at all to 7 = I enjoyed it a lot) [options 1–7]

Q12. As a listener, how familiar are you with twentieth-century classical music? (from 1 = I am not familiar with it at all to 7 = I am very familiar with it) [options 1–7]

Q13. How often do you listen to twentieth-century classical music? (from 1 = never listen to 7 = listen every day) [options 1–7]

Q14. How familiar are you with the piece performed? (from 1 = I have never heard of it to 7 = I have heard it many times) [options 1–7]

Q15. Does participation in a scientific experiment such as this increase or decrease your enjoyment of a performance? (from 1 = it significantly decreases my enjoyment to 7 = it significantly increases my enjoyment) [options 1–7]

Q14. What motivated you to come to tonight’s event (select all that apply)? [options: I wanted to learn more about music and maths working together, I am a regular attendee of RNCM events, I wanted to take part in a scientific study, a friend/family member asked me to come along]

Q16. Please use this box for any other comments you wish to make [followed by a text box]

## References

[B1] Aljanaki A. Wiering F. Veltkamp R. C. (2015). MediaEval 2015: a segmentation-based approach to continuous emotion tracking. *Paper Presented at the MediaEval* (Utrecht: Utrecht University).

[B2] Asch S. E. (1951). “Effects of group pressure upon the modification and distortion of judgments,” in *Groups, Leadership And Men; Research In Human Relations*, ed. Guetzkow H. (New York, NY: Carnegie Press), 177–190.

[B3] Bigand E. (2002). More about the musical expertise of musically untrained listeners. *Paper Presented at the Conference on Neurosciences and Music: Mutual Interactions and Implications on Developmental Functions*, Venice 10.1196/annals.1284.041

[B4] Bigand E. Poulin-Charronnat B. (2006). Are we “experienced listeners”? A review of the musical capacities that do not depend on formal musical training. *Cognition* 100 100–130. 10.1016/j.cognition.2005.11.007 16412412

[B5] Brandstetter J. Rácz P. Beckner C. Sandoval E. B. Hay J. Bartneck C. (2014). “A peer pressure experiment: recreation of the Asch conformity experiment with robots,” in *Proceedings of the 2014 IEEE/RSJ International Conference on Intelligent Robots and Systems*, Chicago, IL.

[B6] Bregman A. S. (1990). *Auditory Scene Analysis: The Perceptual Organization Of Sound.* Cambridge, MA: Cambridge University Press.

[B7] Broughton M. Stevens C. Schubert E. (2008). “Continuous self-report of engagement to live solo marimba performance,” in *Proceedings of the 10th International Conference on Music Perception and Cognition (ICMPC10)*, Sapporo.

[B8] Bruderer M. J. McKinney M. F. (2008). Perceptual evaluation of models for music segmentation. *Paper Presented at the Proceedings of the 4th Conference on Interdisciplinary Musicology*, Thessaloniki.

[B9] Cambouropoulos E. (2006). Musical parallelism and melodic segmentation: a computational approach. *Music Percept.* 23 249–268.

[B10] Caplin W. E. (2001). *Classical form: A Theory Of Formal Functions For The Instrumental Music Of Haydn, Mozart, And Beethoven.* Oxford: Oxford University Press.

[B11] Chew E. (2001). “Modeling tonality: Applications to music cognition,” in *Proceedings of the 23rd Annual Meeting of the Cognitive Science Society*, New York, NY.

[B12] Clarke E. F. Krumhansl C. L. (1990). Perceiving musical time. *Music Percept.* 7 213–252.

[B13] Cook N. (1987). Musical form and the listener. *J. Aesthet. Art Critic.* 46 23–29.

[B14] Deliège I. Melen M. Stammers D. Cross I. (1996). Musical schemata in real-time listening to a piece of music. *Music Percept.* 14 117–159.

[B15] Dibben N. (1994). The cognitive reality of hierarchic structure in tonal and atonal music. *Music Percept.* 12 1–25.

[B16] Drake C. Bertrand D. (2001). The quest for universals in temporal processing in music. *Psychol. Sci.* 13 71–74. 10.1111/j.1749-6632.2001.tb05722.x 11458828

[B17] Egermann H. Pearce M. T. Wiggins G. A. McAdams S. (2013). Probabilistic models of expectation violation predict psychophysiological emotional responses to live concert music. *Cogn. Affect. Behav. Neurosci.* 13 533–553. 10.3758/s13415-013-0161-y 23605956

[B18] Eitan Z. Granot R. Y. (2008). Growing oranges on mozart’s apple tree: “inner form” and aesthetic judgment. *Music Percept.* 25 397–418.

[B19] Foote J. (2000). Automatic audio segmentation using a measure of audio novelty. *Paper Presented at the 2000 IEEE International Conference on Multimedia and Expo. ICME2000*, London.

[B20] Geringer J. M. (1995). Continuous loudness judgments of dynamics in recorded music excerpts. *J. Res. Music Educ.* 43 22–35.

[B21] Granot R. Y. Jacoby N. (2011). Musically puzzling I: sensitivity to overall structure in the sonata form? *Mus. Sci.* 15 365–386.

[B22] Granot R. Y. Jacoby N. (2012). Musically puzzling II: sensitivity to overall structure in a Haydn E-minor sonata. *Mus. Sci.* 16 67–80.

[B23] Hanninen D. A. (2003). A theory of recontextualization in music: analyzing phenomenal transformations of repetition. *Music Theor. Spect.* 25 59–97.

[B24] Hartmann M. Lartillot O. Toiviainen P. (2017). Interaction features for prediction of perceptual segmentation: effects of musicianship and experimental task. *J. New Music Res.* 46 156–174.

[B25] Jensen K. (2007). Multiple scale music segmentation using rhythm, timbre, and harmony. *EURASIP J. Appl. Signal Proc.* 2007:159.

[B26] Krumhansl C. L. (1979). The psychological representation of musical pitch in a tonal context. *Cogn. Psychol.* 11 346–374.

[B27] Krumhansl C. L. (1996). A perceptual analysis of mozart’s piano sonata K. 282: segmentation, tension, and musical ideas. *Music Percept. Interdiscipl. J.* 13 401–432.

[B28] Krumhansl C. L. Kessler E. J. (1982). Tracing the dynamic changes in perceived tonal organization in a spatial representation of musical keys. *Psychol. Rev.* 89:334. 7134332

[B29] Lerdahl F. (1992). Cognitive constraints on compositional systems. *Contemp. Music Rev.* 6 97–121.

[B30] Lerdahl F. Jackendoff R. (1983). *A Generative Theory of Tonal Music.* London: MIT Press.

[B31] Loui P. Wessel D. L. Kam C. L. H. (2010). Humans rapidly learn grammatical structure in a new musical scale. *Music Percept.* 27 377–388. 10.1525/mp.2010.27.5.377 20740059 PMC2927013

[B32] Marsden A. (2005). Generative structural representation of tonal music. *J. New Music Res.* 34 409–428.

[B33] Meyer L. B. (1956). *Emotion and Meaning in Music.* Chicago: Chicago University Press.

[B34] Miller G. A. (1956). The magical number seven, plus or minus two: some limits on our capacity for processing information. *Psychol. Rev.* 63 81–97. 10.1037/0033-295x.101.2.343 13310704

[B35] Ockelford A. Sergeant D. (2012). Musical expectancy in atonal contexts: musicians’ perception of “antistructure”. *Psychol. Music* 41 139–174.

[B36] Phillips M. Cross I. (2011). “About musical time–effect of age, enjoyment, and practical musical experience on retrospective estimate of elapsed duration during music listening,” in *Proceedings Of The Multidisciplinary Aspects Of Time And Time Perception*, Athens.

[B37] Phillips M. E. (2019). Rethinking the role of the golden section in music and music scholarship. *Creativ. Res. J.* 31 419–427.

[B38] Rohrmeier M. Rebuschat P. Cross I. (2011). Incidental and online learning of melodic structure. *Conscious. Cogn.* 20 214–222. 10.1016/j.concog.2010.07.004 20832338

[B39] Rolison J. J. Edworthy J. (2012). The role of formal structure in liking for popular music. *Music Percept. Interdiscipl. J.* 29 269–284.

[B40] Rothstein W. (1991). On implied tones. *Music Analy.* 10 289–328.

[B41] Schenker H. (1979). *Free Composition (der Freie Satz): Heinrich Schenker.* London: Longman.

[B42] Schulze K. Dowling W. J. Tillmann B. (2012). Working memory for tonal and atonal sequences during a forward and a backward recognition task. *Music Percept.* 29 255–267.

[B43] Sears D. Caplin W. E. McAdams S. (2014). Perceiving the classical cadence. *Music Percept.* 31 397–417.

[B44] Spies B. (2005). Facilitating access to atonal music: ligeti’s second string quartet. *J. Musical Arts Africa* 2 55–69.

[B45] Steinitz R. (1996). The dynamics of disorder. *Musical Times* 137 7–14.

[B46] Steinitz R. (2011). *Gyorgy Ligeti.* London: Faber & Faber.

[B47] Stevens C. J. Tardieu J. Dunbar-Hall P. Best C. T. Tillmann B. (2013). Expectations in culturally unfamiliar music: influences of proximal and distal cues and timbral characteristics. *Front. Psychol.* 4:789 10.3389/fpsyg.2013.00789 PMC3819523 24223562

[B48] Straus J. N. (2016). *Introduction to Post-Tonal Theory.* New York, NY: WW Norton & Company.

[B49] Temperley D. (2007). *Music and Probability.* Cambridge, MA: MIT Press.

[B50] Tillmann B. Bigand E. (1996). Does formal musical structure affect perception of musical expressiveness? *Psychol. Music* 24 3–17.

[B51] Tillmann B. Bigand E. (2004). The relative importance of local and global structures in music perception. *J. Aesthet. Art Critic.* 62 211–222.

[B52] Tillmann B. Bigand E. Madurell F. (1998). Local versus global processing of harmonic cadences in the solution of musical puzzles. *Psychol. Res.* 61 15–17.

[B53] Zajonc R. B. (2001). Mere exposure: a gateway to the subliminal. *Curr. Direct. Psychol. Sci.* 10 224–228.

